# Enhancing community integration after incarceration: findings from a prospective study of an intensive peer support intervention for veterans with an historical comparison group

**DOI:** 10.1186/s40352-022-00195-5

**Published:** 2022-11-08

**Authors:** Justeen Hyde, Thomas Byrne, Beth Ann Petrakis, Vera Yakovchenko, Bo Kim, Graeme Fincke, Rendelle Bolton, Christy Visher, Jessica Blue-Howells, Mari-Lynn Drainoni, D. Keith McInnes

**Affiliations:** 1 Center for Healthcare Organization and Implementation Research, VA Bedford Healthcare System, Bedford, MA, 200 Springs Road, MS 152, Bldg. 70, Rm 285, Bedford, Bedford, MA 01730 USA; 2 General Internal Medicine, Boston University School of Medicine, Boston, MA USA; 3 School of Social Work, Boston University, Boston, MA USA; 4 Center for Health Equity Research and Promotion, VA Pittsburgh Healthcare System, Pittsburgh, PA USA; 5 Center for Healthcare Organization and Implementation Research, VA Boston Healthcare System, Boston, USA; 6 Department of Health Law Policy and Management, Boston University School of Public Health, Boston, MA USA; 7 The Heller School for Social Policy and Management, Brandeis University, Waltham, MA USA; 8 Center for Drug & Health Studies, Department of Sociology and Criminal Justice, University of Delaware, Wilmington, DE USA; 9 VA Healthcare for Re-Entry Veterans, U.S. Department of Veteran Affairs, Washington, USA; 10 Section of Infectious Diseases, Boston University School of Medicine, Boston, MA USA

**Keywords:** Incarceration, Reentry, Peer Support, Veterans, Linkage to Healthcare, Linkage to Housing

## Abstract

**Background:**

The transition to the community after incarceration presents challenges for returning citizens, including the immediate need to secure housing, employment, and income. Additionally, health care is essential for this population due to high rates of chronic physical health and mental health problems and substance use disorders. There is growing recognition of the need for interventions that support returning citizens as they navigate community reintegration while simultaneously tending to physical and behavioral health needs. We developed and pilot tested a peer support intervention designed to provide social, emotional, and logistic support and promote linkage and engagement in healthcare for returning citizens. We tested the intervention with US military veterans in Massachusetts who were being released from prison and jail. Outcomes related to linkage to and engagement in healthcare were evaluated using an historical comparison group. Engagement in peer support, housing status, and reincarceration rates were monitored for the intervention group.

**Results:**

There were 43 veterans in the intervention group, and 36 in the historical comparison group. For linkage to primary care within 90 days of release, there were no statistically significant differences between the intervention and comparison groups (58% versus 67%). Intervention participants were significantly more likely to receive substance use treatment than the comparison group (86% versus 19%, *p* < .0001) and the mean monthly substance use visits was greater in the intervention group (0.96 versus 0.34, *p* < .007). Engagement in mental health services was greater for the intervention group than the comparison group (93% versus 64%, *p* < .003). There were no significant differences between groups for emergency department use and hospitalization. At the end of the study period, the majority of intervention participants who had been released for over a year were living in permanent housing (84%). Recidivism among the was low, with 7% re-arrested during the study period.

**Conclusions:**

Augmenting reentry support through intensive peer support appears to have substantial benefits for veterans in terms of engaging them in health care and contributing to their longer-term stability, including housing and recidivism. Flexible reentry support such as this intervention may be well suited to meet the widely varying needs of returning citizens.

## Introduction

The transition from prison or jail back to community settings poses a number of challenges for individuals who are reentering society following a period of incarceration (hereafter referred to as ‘returning citizens’). Safe housing and income are among the most immediate and basic needs (Western, 2002; Visher & Travis, 2003; Fontaine & Beiss, 2012; Couloute, 2018). Prior research has also called attention to the disproportionately high rates of chronic physical and mental health conditions, and substance use disorders among individuals with incarceration experience in comparison to the general population (Mallik-Kane et al., 2008; Binswanger, 2007; Wilper et al., 2009; Williams et al, 2010; Fox et al., 2014; Finlay et al., 2016, 2019; Fazel and Baillargeon, 2011). These health conditions increase the risk of adverse outcomes upon or shortly after return, including unemployment, homelessness, criminal behavior, and premature death (Baillargeon et al., 2010; Binswanger et al., 2007, 2012; Couloute, 2018; Kinner & Young, 2018; Tsai et al., 2014; Wang et al., 2013; Whipple et al., 2016; Wortzel et al, 2012;). In acknowledgement of these risks, there is growing recognition of the need for interventions that support returning citizens as they navigate the process of integrating back into society while simultaneously attending to physical and behavioral health needs (Freudenberg, et al, 2005; Marlow et al., 2010; Binswanger et al., 2011, Patel, Boutwell, Brockmenn and Rich, 2014; Vail, Niyogi, Henderson & Wennerstrom, 2017).

Ideally, interventions to support returning citizens begin at intake into a correctional facility and continue beyond the initial reentry period to ensure long term success (Dumont et al., 2012; La Vigne, et al., 2008). There is a wealth of research demonstrating disparities in health, mental health, and socio-economic status among returning citizens post-incarceration compared to their non-incarcerated peers (La Vigne, et al., 2008; Dickman, Rich and Wakeman, 2011; Dumont et al., 2013; Liptak, 2016). While attending to these issues during incarceration could help increase the chances of success post-release, reentry planning is highly variable from state to state, and from one correctional facility to another. Often, reentry planning focuses on immediate needs at the moment of release, such as transportation out of a facility and initial housing. Few returning citizens are provided with planning around health and mental health needs or are actively linked to care upon release (Fox et al., 2014; Wang et al., 2008). Lack of health insurance, competing economic needs, residential instability, and unaddressed mental health and substance use needs are significant barriers to accessing health and social services after returning to the community (Mallik-Kane, Paddock and Jannetta, 2018; Marlow et al., 2010; Vail et al., 2017). Less often highlighted are challenges related to inexperience with routine healthcare and negative prior experiences with social services (Hyde et al., 2021), which may in part underlie reasons for limited engagement in physical or behavioral health care in the months following release (Bellamy et al., 2019; Mallik-Kane & Visher, 2008; O’Connell et al., 2020).

The challenges faced upon reentry have multiplying effects. For example, poor health impedes one’s ability to gain employment, which reduces the likelihood of securing safe, long-term housing, which is key for successful community reintegration. Preventing or interrupting returning citizens’ downward spiral requires a coordinated system of support, yet few evidence-based interventions for returning citizens focus on linkage and connection to both healthcare and support services after release (Kendall, et al., 2018; Visher, et al., 2017). Therefore, returning citizens may prioritize obtaining housing and income over physical, mental health, and substance use treatment needs. One of the few programs directed at increasing access to and engagement in healthcare post-incarceration is the Transitions Clinic Networks, a group of 17 healthcare clinics that focus on the healthcare needs of returning citizens and include case management provided by a community health worker. These clinics, importantly, have been shown to reduce emergency department utilization among the returning citizens they serve (Wang et al, 2012). However, prior research has demonstrated limited evidence of sustained engagement in primary or other outpatient care to manage chronic conditions. (Binswanger et al, 2011; Fox et al., 2014; Hunter, et al., 2016; Richie, 2001; Shavit et al., 2017; Wang et al, 2010, 2012).

Interventions that include consistent, tangible support to help navigate the myriad challenges that returning citizens face as they work towards social reintegration are needed (Goldstein et al., 2009; LeBel, 2007; Rowe et al., 2007). There is a small body of evidence indicating that forensic peer specialists may be ideally suited to provide this kind of support (Bellamy et al., 2019; Rowe, et al., 2007; Davidson et al., 2009; Barrenger, Hamovitch, Rothman, 2019). Forensic peer specialists are individuals who have personal experience with mental health and/or substance use problems and are knowledgeable about the criminal justice system (Adams & Lincoln, 2020). They have navigated many of the challenges that returning citizens face and serve as an inspirational reminder that recovery is possible (Davidson, et al., 2012; Barrenger et al., 2019). Similar to other peer specialists, forensic peers can provide a broad range of assistance that includes emotional support (e.g., encouragement to work through frustrations and challenges, someone to talk with when difficulties arise), physical support (e.g., transportation to and from service or healthcare agencies, assistance filling out paperwork), and role modeling (e.g., demonstrating how to set up a bank account, how to make a healthcare appointment) (Chinman et al., 2014; Bellamy et al., 2019; Reingle, et al., 2019; Shalaby & Agyapong, 2020;). They can also help facilitate connection to trusted resources and services, drawing on their personal experience and returning citizens’ own values and preferences (Davidson et al., 2012).

We aim to contribute to the growing evidence base around forensic peer specialists by presenting findings from a pilot intervention developed within the U.S. Department of Veteran Affairs (VA) to improve linkage and engagement in healthcare and social support services among military veterans who are released from prison or jail in one Northeastern state. The Post-Incarceration Engagement (PIE) intervention uses peer specialists to enhance existing reentry services offered by the VA. Here we examine the implementation and outcomes of this intervention, with the overarching goal of better understanding the feasibility and potential impact of the PIE peer support intervention on health care, housing and criminal justice outcomes. Specifically, our aims are to: 1) describe the volume and type of activities of peer specialists (feasibility); 2) compare linkages to VA health care immediately following release between PIE participants and a historical comparison group of re-entering Veterans who did not participate in the intervention (linkage to care); and 3) describe rates of linkage to stable housing and recidivism among intervention participants (impact). The first objective provides important information about the feasibility of the intervention, whereas the latter two provide information about its potential impact.

### Description of post-incarceration engagement intervention

The Post-Incarceration Engagement (PIE) intervention is a peer-based enhancement to the VA Healthcare for Reentry Veterans (HCRV) program (Finlay et al., 2017). The HCRV program launched in 2007 and consists of one to two outreach specialists per state who meet with incarcerated veterans and assist them with reentry planning, including finding housing and setting up appointments within the VA health system. Once a veteran is released, outreach specialists generally have limited capacity to provide extensive assistance to support them with reentry plans. The PIE intervention fills this gap by incorporating peer support specialists into the HCRV program to extend the provision of social and logistical support for approximately 6 months post-incarceration. PIE peer specialists (“PIE peers” hereafter) are veterans with life experiences similar to the veterans they serve in the intervention.

**Fig. 1:**
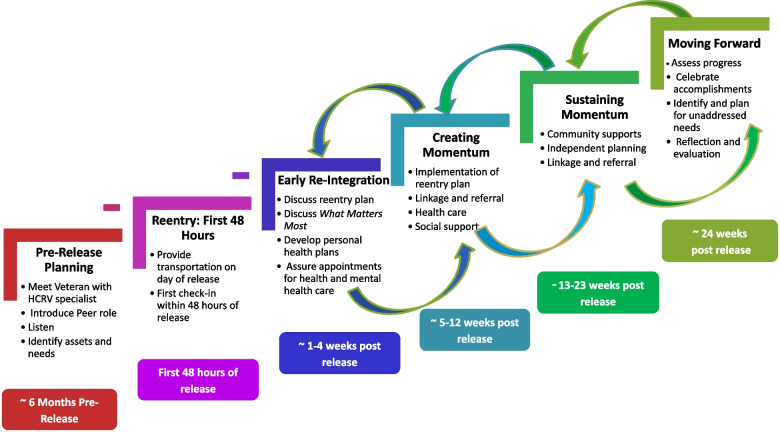
Overview of post-incarceration engagement activities

The PIE intervention was developed by the authors following a qualitative formative phase, which included interviews with veterans with recent incarceration experience, reentry specialists and peer specialists within the VA and in the community, and state Department of Correction representatives (Hyde et al., 2021; Kim et al, 2019; Simmons et al., 2017). Findings from our previously published formative evaluation were used to develop core components of the PIE intervention and an intervention guide to orient peer specialists to the purpose and structure of the work. The Post-Incarceration Engagement guide outlined an overview of reentry and reintegration needs, the HCRV program and purpose of adding peer support specialists to the team, training recommendations, core roles of peer specialists and the types social, emotional, and logistical support they should be prepared to provide throughout the processes of reentry and social integration. Figure 1 provides an overview of the peer support approach.

The work of PIE peers ideally begins pre-release, supporting the HCRV outreach specialist with discharge planning and relationship building. The intensive work typically begins on the day of release, with peers providing tangible support, such as transportation to housing and parole or probation offices. In the ensuing six months, PIE peers provide a range of social and emotional support, linkage and referral to healthcare and social services, and role modeling of life skills. The intervention draws on a Whole Health approach to care, which is a growing movement within the VA healthcare system to move from a disease-centric to a whole person model of care (Bokhour et al., 2020a, 2020b(a); Bokhour et al., 2020a, 2020b (b); Purcell et al., 2021). In practice, this means beginning care with a shared understanding of what matters most to individuals and what they want their health for and allowing this understanding to guide how and what care is provided. Applied to the PIE intervention, the Whole Health approach includes guided discussions with participants to learn what matters most in their lives and what they would want their lives to be like post-incarceration. These conversations serve as a springboard for developing short and long-term goals and action plans. Peers support the pursuit of these goals along with making referrals and linking veterans to medical, social and other types of services, providing emotional support and encouragement, and role modeling life skills.

The pilot intervention included 2 PIE peers who were trained by project team members, including an anthropologist (JKH), a public administration specialist (BAP), and a public health specialist (DKM). PIE peers received clinical supervision from the HCRV outreach specialist, a licensed independent clinical social worker. The evaluation examined data from the operation of PIE between December 2017 and September 2019.

## Methods

The goal of the pilot study was to assess the feasibility and potential impact of the PIE intervention. We collected data that allowed us to assess the activities of peers and the healthcare, housing, and criminal justice-related outcomes of interest to our study aims. The pilot was submitted to the Institutional Review Board (IRB) at the VA Bedford Healthcare System (Bedford, Massachusetts, USA), which determined it was a quality improvement project as per VA handbook 1200.05. The need for continued IRB review was waived.

### Setting

Persons convicted of felony offenses are placed in 1 of 16 prisons run by the Massachusetts Department of Correction or 1 federal prison. Individuals who are convicted of misdemeanors with sentences ranging from one day to two and a half years are committed to 1 of 14 county-run houses of correction. The Healthcare for Reentry Veterans program involved in the pilot study serves a catchment area that includes 4 state prisons, 1 federal prison and 3 houses of correction. Although outreach is provided to support reentry planning with Veterans who are incarcerated in this catchment area, peer support during the pilot study focused on Veterans who were released to communities served by the program’s VA Medical Center. Total releases from all Departments of Correction during the study period was 3,803 individuals, approximately 21% or 799 of which were released to communities in the two counties served by the VA Medical Center (Cannata et al., 2021). With an estimated 5.5% of the Massachusetts population being Veterans (U.S. Census, 2022), an estimated 44 Veterans serving time in Massachusetts Department of Correction facilities were likely released to communities in the catchment area during the study period. However, not all Veterans are eligible for VA health care services. Criteria include having an honorable discharge, combat experience, injury during service, and income, among others. There is no equivalent data for individuals released from County Houses of Correction, limiting our ability to estimate the total number of Veterans released from a Department of Correction or House of Correction facility who could have potentially been served by PIE peers.

### Sample

Criteria for inclusion in the PIE intervention included being a U.S. military veteran, eligible for release from incarceration in 6 months or less or newly released (up to 3 months), and eligible for VA health services (e.g., served in the active military, naval or air service, separated under any condition other than dishonorable). The VA’s Network Homeless Program Office for New England maintains a regional database of incarcerated veterans. The database includes information about eligibility for VA healthcare services and anticipated release dates. Information about veterans with upcoming releases (6 months or less) are regularly provided to the HCRV case manager. During the study period, the HCRV case manager introduced veterans with upcoming releases to the PIE intervention. For veterans who expressed interest after the introduction, the PIE peer made every attempt to have an in-person meeting prior to the scheduled release date. In some cases, the first in-person encounter did not happen until the day of release or beyond. Veterans who were undecided about, or refused participation were provided with contact information and informed that a PIE peer could work with them if they changed their mind. Veterans who did not participate continued to receive standard release planning and care provided by the correctional system to all returning citizens.

Participants in the study were enrolled between November 1, 2017 and September 30, 2019. The historical comparison group comprised veterans who entered the HCRV program and had a valid release date indicating they were released from a house of correction or prison in Massachusetts between January 1, 2016 and October 31, 2017 (the period immediately preceding the launch of the PIE intervention) (*N* = 36). The standard of care for release planning and assistance remained the same from 2016 through the end of the study period in 2019. This comparison group was identified using administrative data from the HCRV program, obtained from the VA Homeless Operations Management and Evaluation System (HOMES) database, which is designed to track utilization of VA specialized homeless programs and Veterans Justice Programs, including HCRV.

### Measures

#### Demographic information

Demographic information for pilot study participants was obtained from a baseline intake form that included information about age, gender, race and ethnicity, marital status and self-rated health, and total length (in months) of current episode of incarceration. For the historical comparison group, we obtained limited demographic data from the Homeless Operations Management and Evaluation System (HOMES) assessment form, which is typically completed by an HCRV Outreach Specialist, and which also includes information about length of current episode of incarceration. Both the study intake form and HOMES assessment used a single item Self-Rated Health question that asks people how healthy people think they are, with 5 response options to choose from that range from poor to excellent (Ware & Sherbourne, 1992).

#### Activities of PIE peers

Information regarding the type, content and duration of encounters that PIE peers had with participants were initially recorded by peers on paper forms using open ended fields. Encounters refer to interactions between a peer and a veteran or interactions a peer has with a third party (e.g. community housing program) on behalf of a veteran. An encounter could entail two or more types of activities, for example social/emotional support and accompaniment to a medical appointment. One year into the intervention, the study team reviewed and coded the encounter data and used the findings to create a more structured encounter form, which was more time efficient for peers to complete. This form captured the date, duration and type of activities engaged in during encounters (e.g. transportation to appointments, social and emotional support). This form was programmed in REDCap® (Harris et al., 2009), a secure, web-based application to support data capture for research studies and was completed by the peers after each encounter. Information from paper encounter forms was also transferred into the REDCap® database. There are no encounter data for the historical comparison group because they did not have a PIE peer working with them.

#### Linkages to VA health care following release

We used VA administrative data sources to construct measures of linkages to an array of VA health care services within 90 days of release from incarceration. Specifically, we used data from the VA’s Corporate Data Warehouse (CDW)—a national repository of information from VA’s electronic medical records—to assess, for the first 90 days after release: 1) any use of primary care, outpatient substance use treatment, outpatient mental health, other outpatient care, emergency department, or inpatient treatment; 2) length of time from release to first episode of utilization of each one of these services; and, 3) the number of episodes for each type of service. We used clinic stop codes, which indicate the type of clinic in which a particular outpatient visit occurred to categorize outpatient visits as being for primary care, substance use treatment, outpatient mental health, outpatient care or emergency department. For substance use and mental health visits, both individual and group appointments (e.g. group counselling) were included. The outpatient visits for mental health and substance use may include outpatient visits in which medication was prescribed, although we do not directly capture these prescriptions and whether they were actually filled by a VA pharmacy. For inpatient treatment, we assessed any inpatient hospitalizations within 90 days of release. We also used specialty care codes to create categories for two types of inpatient hospitalizations: mental health/substance use related and medical.

#### Housing and recidivism outcomes

We captured information about only PIE participants’ housing status in follow up interviews conducted at 3- and 6-months post-release. The project manager (BAP) also maintained an Excel® spreadsheet that was used to keep track of transitions in housing post-release, with information pulled from follow up interviews and supplemented with information provided by veterans and/or PIE peers. To obtain information about housing status at the end of the study period (September 2019), the project manager reached out directly to veterans to inquire about their current housing and recorded it on the spreadsheet. For the few veterans she could not reach, she obtained last known address information from the medical record.

Recidivism is defined for the purposes of this study as any criminally sentenced individual released to the community who is re-incarcerated for a new sentence or violation of parole or probation to a Massachusetts state, county or a federal facility. Information about rearrest and reincarceration was obtained from the HCRV specialist and PIE peer through weekly debrief meetings with peer and quarterly meetings with the HCRV specialist. PIE Peers aimed to have at least weekly contact with Veterans during the intervention period, which enabled the team to maintain good situational awareness of criminal justice involvement. The team also had strong relationships with local parole officers and were informed of legal concerns or challenges when they arose. Information about rearrest and reincarceration was recorded in the same outcomes spreadsheet used to record housing status (noted above). Comparable information about housing status and reincarceration one-year post-release was not available for the historical comparison group.

### Analysis

Analysis proceeded in three phases, in line with our three study aims. We used descriptive statistics to summarize the volume and intensity of peer activities in support of PIE participants (Aim 1). To examine access to and engagement in care outcomes (Aim 2), we compared our measures of linkages to VA health care following release between PIE participants and our historical comparison group. Specifically, we used bivariate tests (Chi-Square/Fisher Exact Tests and t-tests, as appropriate) to assess the relationship between PIE participation and linkages with VA health care. Finally, we examined housing and reincarceration outcomes for intervention participants (Aim 3) by abstracting data from the participant database and assigning each participant to one of five housing types that best matched their current status. Any history of involvement with the criminal justice system during the study period was also abstracted. Given the rolling enrollment of participants over the two-year study period, we examined housing outcomes by length of time since release. To account for differences in housing status related to variation in length of time since release and the end of the study period, we organized participants into two groups: those released from incarceration less than a year before the end of the study period (September 30, 2019) and those released a year or more at the end of the study period. Housing disposition and recidivism is presented for each group in Table 5. We did not have access to comparable data on housing and reincarceration for the comparison group.

## Results

A total of 43 Veterans engaged with PIE Peers post-release. An additional 5 Veterans received peer support on the day of release (e.g., transportation to probation/parole and housing) but declined further peer services. Only 5 Veterans declined any peer services and 4 Veterans expressed interest in working with a PIE peer while incarcerated but never received services post-release. Table 1 provides demographic information for Veterans who engaged in the intervention and comparison groups. Participants were on average 49.3 years of age (range 28–72). Veterans in the historical comparison group (*n* = 36) averaged 53.8 years (range 28–86). All participants in both groups were male and both groups were predominately White/Caucasian (72% in the PIE group and 83% in the historical comparison group; *p* = 0.452). There were no statistically significant differences between the groups with respect to age (ns, *p* = 0.109), sex (*p* = 1.0), race (*p* = 0.452), ethnicity (*p* = 1.0), or marital status (*p* = 0.280). Similarly, while the length of the most recent episode of incarceration was shorter for the intervention group relative to the comparison group (47.7 months vs. 76.6 months) this difference was not statistically significant (*p* = 0.072). Finally, there were no significant differences (*p* = 0.144) in self-reported health status between the two groups, with approximately one-third in each group rating their health as Excellent or Very Good and similar proportions rating their health as Good.

**Table 1:** Returning citizen characteristics

	PIE Group (*n* = 43)		Comparison Group (*n* = 36)		
	n	%	n	%	*p*-value
Age, mean (SD)	49.3 (13)	range 28–72	53.8 (14)	28–86	.109
Gender, male	43	100%	100%		1.00
Race					.452
African American or Black	10	23%	6	17%	
White	31	72%	30	83%	
Multiple/Other/Declined	2	5%	0	0%	
Ethnicity, Hispanic	2	5%	1	3%	1.00
Marital status					.280
Not partnered^a^	37	86%	34	94%	
Partnered^b^	6	14%	2	6%	
Self-rated health					.144
Excellent	6	14%	8	22%	
Very good	9	21%	3	8%	
Good	17	40%	12	33%	
Fair	8	19%	5	14%	
Poor	3	7%	4	11%	
Missing	0	0%	4	11%	
Length of most recent incarceration in months, mean (SD)	47.7 (63.8)		76.6 (77.2)		.072

^a^ Single, divorced, separated or widowed; ^b^ Married or with partner

*PIE* Post Incarceration Engagement, *SD* Standard deviation

### Activities of PIE Peers

Tables 2 and 3 provide details about the frequency and content, respectively, of peer support services provided to participants. There were 435 encounters recorded, and the majority (92%) were provided post-release (Table 2). Encounter length varied, with an average of 138 min and ranging from a few minutes (2 min) to 10 h (600 min). Pre-release encounters occurred with 19% (*n* = 8) of the veterans served, lasting an average of 3.3 h (210 min; range from 60–360 min). Nearly half (47%) of participants had an encounter on the day of release, which lasted an average of 357 min (range 90–630 min).

**Table 2:** Frequency of PIE Peer Encounters

	encounters	% of tot	RCs #	RCs %	Avg minutes	sd	min	max
Pre-release	15	3.4%	8	19%	210	125	60	360
Day of release	20	4.6%	20	47%	357	134	90	630
Post release	400	92%	43	100%	138	116	2	600
TOTAL	435		43					

*RC* Returning citizen, *Avg* Average, *sd* Standard deviation, *tot* Total

**Table 3:** Description of PIE Peer Encounters, intervention participants only

Encounter Type	RCs (*N* = 43)	% of RCs
**Pre-release**	**8**	**19%**
Pre-release paperwork or information gathering	6	75%
Social and emotional support	6	75%
Verify reentry plan (with parole, probation, facility reentry specialist, etc.)	5	63%
Appointment preparation (documents, applications, etc.)	4	50%
Meetings with collateral partners on participants behalf	2	25%
**Day of release**	**20**	**47%**
Social/emotional support	20	100%
Transportation to housing/legal stipulations (e.g. to probation and/or parole)	19	95%
Obtain basic clothing and supplies (e.g. toiletries, food, cell phone)	12	60%
Support connecting with family/friends	7	35%
Financial (cashed check, banking, etc.)	5	25%
Medical appointment (set-up or saw medical or mental health provider)	4	20%
**Post release**	**43**	**100%**
Social/emotional support	43	100%
Transport	38	88%
Transport—Legal appointment	24	56%
Transport—Non-VA benefits	17	40%
Transport—Financial (open bank account, cash or deposit checks, etc.)	16	37%
Linkage to concrete services or resources	39	91%
Appointment Preparation (paperwork, appointment verification and/or reminder)	35	81%
Accompany—any appointment/errands	28	65%
Support connecting with family/friends	23	53%
Acclimate to new community and navigate VA campus and healthcare system	20	47%
Worked on PIE-related forms with Veterans	19	44%
Skill building	19	44%
Basic needs: Made arrangements for or gave Veteran clothing, toiletries, shoes, etc	18	42%
Obtain documents	18	42%

*PIE* Post-Incarceration Engagement, *RC* Returning citizen

Table 3 provides a summary of peer activities, organized by pre-release, day of release, and post-release encounters. During pre-release encounters, 75% of participants received social and emotional support – including descriptions of the resources that would be available to them once released and encouragement that they would make a successful transition. Peers also helped 75% of participants complete paperwork for housing and other resources post-release. For nearly two-thirds of participants (63%), peers also served as a liaison between participant and reentry specialists or parole/probation officers to communicate information relevant to their case.

On average, day of release encounters were the longest, averaging nearly 6 h. All encounters included some social and emotional support. Most (95%) included transportation from the penal facility to their residence. Integrated into transportation is the opportunity for participants to be in a safe place, with peers providing support and encouragement before making their next step back into society. Peer specialists accompanied participants to their first meetings with parole or probation officers and those with sex offenses were driven to police station within their community of residence to register. In addition, peers provided about 60% of participants with seasonal clothing, shoes and toiletries prior to entering into their housing placement. For 20% of participants, peers assisted them in getting immediate medical services.

Peers provided social and emotional support during all post-release encounters. They assisted nearly all participants (91%) with linkage to concrete services or resources and provided transportation (88%) to access them. During the post-release period, most participants (81%) also received assistance from peers with preparing for upcoming appointments by helping to complete paperwork, verify appointments, and write out important questions they want to discuss. Approximately two-thirds (65%) of participants also had a peer accompany them to one or more appointments. Roughly half the veterans received support connecting to family members and friends (53%), acclimating to and navigating VA services (47%), or skill building (44%).

### Linkages to healthcare following release

Table 4 compares rates of linkage to and engagement in care between the intervention and comparison groups within 90 days of release. A majority of veterans had engaged in primary care (58% for intervention and 67% for comparison groups), with no statistically significant differences between the groups (*p* = 0.49). Participants in the PIE intervention were significantly more likely to receive substance use treatment than the comparison group (86% vs 19%, *p* < 0.0001) and the mean number of monthly substance use visits was greater in the intervention group than in the comparison group (0.96 versus 0.34, *p* < 0.007). Engagement in mental health services, similarly, was greater for the intervention group than the comparison group (93% versus 64%, *p* < 0.003). Engagement in other outpatient care was significantly higher for the intervention group than the comparison group on all 3 measures: percent receiving care in the first 90 days (97.7% vs 83.3%, *p* < 0.007), mean time to first visit (0.21 versus 0.66 months, *p* = 0.049) and mean number of visits (2.2 versus 0.78 per month, *p* < 0.001). We did not find significant differences between groups for use emergency department services (*p* = 1.0). Similarly, the 11.6% of Veterans in the intervention group who had any inpatient hospitalization within 90 days did not significantly differ from the 8.3% of the comparison group who did (*p* = 0.72). When differentiating inpatient stays by type, similar findings emerged: 4.6% of the intervention group had a mental health or substance use related hospitalization which did not differ significantly from the 0% of the comparison group (*p* = 0.50) and 7.0% of the intervention group had a medical inpatient hospitalization, which likewise did not significantly differ from the 8.3% of the comparison group who had a medical inpatient hospitalization (*p* = 1.0).

**Table 4:** Comparison of PIE intervention veterans and matched historical comparison group, considering service use within 90 days of release

	Intervention (*N* = 43)	Comparison (*N* = 36)	*p*-value
***Outpatient Linkage***			
**Primary care**			
% With Primary care linkage	58.1%	66.7%	0.49
Primary care linkage (mean time in days to first PCP visit post-release)^1^	24.6	21.6	0.492
PCP visits per month (mean)^1^	0.21	0.17	0.683
**Substance use disorder (SUD) care**			
% with SUD care linkage	86.0%	19.4%	< 0.0001
SUD care linkage (mean time in days to first visit post-release if SUD diagnosis)^1^	15.3	27.6	0.360
SUD visits per month (mean)^1^	0.96	0.34	.007
**Mental health (MH) care**			
% with MH care linkage	93.0%	63.8%	0.003
MH care linkage (mean time in days to first visit –post-release, if MH diagnosis)^1^	18.0	25.2	0.112
MH visits per month (mean)^1^	0.74	0.82	0.100
**Other outpatient care (e.g., endocrinology, cardiology)**			
% with other outpatient care	97.7%	83.3%	0.007
Other outpatient care linkage (mean time in days to first visit –post-release, if MH diagnosis)^1^	6.3	19.8	0.049
Other outpatient visits per month (mean)^1^	2.2	0.78	0.0005
***VA Emergency Department (ED) use***			
% with VA ED use	7.0%	8.3%	1.00
ED visits per month (mean)^1^	0.06	0.06	1.00
***VA Inpatient Hospitalizations***			
% with VA Inpatient Hospitalization use	11.6%	8.3%	0.721
Inpatient episodes per 1 month (mean)^1^	1.8	1.4	0.863

1 = among Veterans with any service use

*PCP* Primary care provider, *HCRV* Health Care for Reentry Veteran, *MH* Mental health

### Linkage to housing and recidivism

Table 5 provides details of the housing disposition of PIE participants at the end of the study period. Among the 24 PIE participants who had been released less than one year before the end of the study period, 25% were living in permanent housing either alone, with a partner, or with a family member. The majority were living in more temporary housing, including transitional housing programs (25%), short-term emergency housing programs (21%) or residential treatment programs (25%). Among the 19 PIE participants who had been released more than a year before the end of the study period, the majority (85%) had moved into permanent housing. One participant was still in transitional housing (5%), one was in a residential mental health program (5%), and one had died after completing the PIE program (5%). A total of three PIE participants (7%) were arrested at some point during the study period, two for technical parole violations and one for Driving Under the Influence. The latter was incarcerated at the end of the study period.

**Table 5:** Disposition of PIE participants at study end for housing, health, and incarceration, *n* = 43

	Released < 1 year as of end of study period (*n* = 24)	Released ≥ 1 year as of end of study period (*n* = 19)
	No. (%)	No. (%)
Permanent housing^a^	6 (25)	16 (84)
Transitional housing^b^	6 (25)	1 (5)
Short-term emergency housing^c^	5 (21)	0 (0)
Residential Treatment Program^d^	6 (25)	0 (0)
Street homeless/unsheltered	0 (0)	0 (0)
Hospitalized		1 (5)
Incarcerated^e^	1 (4)	
Deceased	0 (0)	1 (5)

*PIE* Post Incarceration Engagement

^a^ includes HUD-VASH and other permanent apartment/home or living with family

^b^ includes transition-in-place and transitional rehabilitation residence programs

^c^ includes GPD temporary bed and shelter

^d^ includes VA Domiciliary and other residential treatment programs

^e^ 2 additional Veterans were re-incarcerated in the < 1 year period, but they were released as of the end of the study period

## Discussion

In one of the few studies of forensic peer support, we found evidence that our 6-month intensive initiative was feasible to implement and contributed to returning citizens’ greater linkage and engagement with substance use, mental health, and other specialty care than a historical comparison group. Rates of permanent housing one-year post-release were high and reincarceration low in the intervention group. Though there were no data to assess this in the comparison group, the rates of reincarceration following the intervention are better than the state average (7% vs 17%) (Papagiorgakis, 2018; Cannata, et al., 2021).

Our study is also notable for the detailed documentation of how peers spent their time assisting returning citizens, including considerable social and emotional support at each of the three phases we examined – pre-release, day of release, and post release. PIE peers often helped returning citizens fulfill their conditions of release by providing transportation to probation and parole appointments. Other common assistance included linkage to services, assisting with appointment preparation, and transporting and accompanying returning citizens as requested to legal, medical, benefits, and other appointments. These types of intensive, social and logistical supports were critical to the success of the intervention. Many returning citizens have few if any relationships with family members to provide support after return, and those that exist are often fraught or frayed due to the strains that incarceration (and the behaviors that led to incarceration) may have placed on them (Western, 2018; Western et al., 2015).

These findings compare favorably with other interventions to support returning citizens, such as the Transitions Clinics model that combines post-incarceration oriented health care clinics and community health workers (Anderson-Facile, 2009; Fox et al., 2014; Morse et al., 2017), and those that use a Critical Time Intervention (CTI) approach (Doleac, 2019; Hignite & Haff, 2017; Hopkin et al., 2018; Lattimore et al., 2010; Malta et al., 2019; Moore et al., 2020). Wang et al.’s (2012) study of Transition Clinics found, in a randomized trial of 200 returning citizens, that while returning citizens’ use of Transition Clinics was associated with lower rates of emergency department (ED) utilization, compared to the control group, there were no differences between groups in utilization of primary care. Our study found no difference in VA emergency department utilization or in VA primary care use between our two groups. However, our positive findings related to engagement in substance use disorder treatment and mental health care (areas not assessed by Wang et al., 2012). Intervention participants had greater likelihood of a substance use disorder (SUD) treatment visit and a mental health (MH) visit in the first 3 months post-release than the comparison group. Additionally, intervention participants had, on average, a greater total number of SUD visits per month than those in the comparison group. This outcome may represent the peers’ first-hand knowledge of the importance of SUD and MH care for the recovery process and the achievement of other life goals and have given them special urgency to guide participants to these services. Primary care, in contrast, is essentially the gateway to most VA services and it is reasonable that rates of primary care utilization were similar between the two groups.

CTI approaches, including programs with intensive case management, also show promise. In their review of interventions to support the transition of individuals living with mental illness back to community settings after a period of incarceration, Hopkin et al., (2018) highlighted several interventions with improved outcomes related to connection to mental health and other services. For example, a study from Washington State, which examined a program consisting of elements of CTI, reported that returning citizens involved in the state’s intensive mental health case management transition program were linked to mental health care more quickly (2.3 days versus 185 days) and had more hours of mental health care in the first three months (92 h versus 5.5 h) than a matched sample (Theurer & Lovell, 2008). In a separate study, the Jail In-Reach project out of Harrison County, Texas found that a comprehensive assessment of physical, mental, and social health needs while incarcerated coupled with case management on the day of release significantly improved linkage with health services among individuals with mental illness (*p* < 0.001) (Buck et al., 2011). Outside the US, there have also been promising studies such as a randomized trial in England of CTI for male RCs with serious mental illness (Shaw 2017). The intervention involved case-manager supported linkage of returning citizens to mental health and substance use treatment, money management, and life-skills training. RCs in the CTI group had greater engagement with community mental health teams (a care coordinator, a care plan, and medical treatment) than the control group at 6 weeks (53% versus 27%, *p* = 0.12). Thus, while interventions have used similar “navigator” or case-manager type approaches to supporting engagement in healthcare and community integration more broadly, the published literature on the use of peer specialists in this role is sparse.

Our efforts to carefully document the peers’ intervention activities, and our regular communications the HCRV specialist, and the intervention participants has provided important insights into the value of consistent social, emotional and logistical support throughout the different phases of community reentry and reintegration. It is likely that there is not one single aspect of peer support that led to the outcomes we found. Rather, we conjecture that it is the combination of support – assistance facilitating veterans’ making and keeping health- and housing-related appointments, logistical support to accomplish important community reintegration tasks, and social and emotional support to weather feelings of anxiety, frustration, and disappointment – that helped participants take critical steps towards engaging in services and securing permanent housing.

Our study suggests the need for interventions that are tailored to the unique needs and circumstances of each returning citizen. The average number of encounters per participant ranged from 1 to 58, with an average of 10. The wide range reflects the flexible structure of the intervention that allowed peers to calibrate the support they provided participants, based on individual needs and the different phases of reentry. Unlike many peer-support and case management programs, PIE peers spent a considerable amount of time “in the field” with veterans, assisting them with community reintegration, building relationships, attending legal, housing, and health appointments with them, and providing social support and encouragement. The ability of peers to provide transportation and other logistical support to help secure and prepare documents (e.g., open bank account), comply with legal stipulations for conditions of probation/parole, and troubleshoot challenges when they arose (e.g., inappropriate housing match) was probably also critical, especially during the first days and weeks of reentry. Peers found, as we have noted in a prior publication from this study (Hyde, et al., 2021), that the intensity of work coincided with major transitions – such as in housing, employment, and relationships.

### Limitations

There are a number of limitations to this work. First, and most importantly, limitations associated with our use of a historical comparison group mean that the associations between participation in the PIE intervention and the service use outcomes we examined should not be considered causal in nature. While the PIE intervention and historical comparison groups were comparable on a number of demographic characteristics and overall self-rated health, we did not have access to more detailed information about each group’s underlying health or behavioral health conditions nor about the complete nature and extent of their prior history of involvement in the criminal justice system. All of these factors as well as secular ones may be confounded with the outcomes we considered. Moreover, we could not account for time-varying contextual factors (e.g. changes in policy, availability of services) that may explain the differences we observed between the groups. As such, any differences we identified between the two groups should be interpreted cautiously, and future research in this area would be improved with a prospective study design with random assignment to the intervention, either at the individual or site level. Second, the intervention was tested in one state, in a large integrated healthcare system, with PIE peers primarily supporting one VA program. Thus, the findings may not generalize to other states or other healthcare settings. Third, there was likely an under-reporting of peer encounters throughout the study period. Documentation of peer services is a challenge. In part this is because of the peers’ need to spend considerable time out in the field providing support to highly vulnerable veterans, which leaves them with limited time for documentation in the medical record. This may also be because the use of computers for word-processing may not be a developed skill for some peers. More efficient and user-friendly ways of documenting peer encounters is needed in order to more fully understand the array and intensity of services provided. Finally, the VA administrative data sources that were used for the historical comparison do not capture appointments or treatment outside of the VA system, thus, for example, there may be undercounting for ED or specialty care visits to a community hospital.

## Conclusions

Augmenting reentry assistance through the use of an intensive peer support intervention appears to have substantial benefits for veteran returning citizens in terms of engaging them in health care and contributing to their longer-term stability, including in regard to housing and recidivism. Larger scale studies are needed, with prospective, randomized designs where possible. Such studies should also include cost components to contribute to an understanding of cost effectiveness of intensive peer support compared to other interventions such as the use of social work case managers who may have more training but are less able to spend time in the field and may not engender the same trust and credibility as peers who have similar lived experience to the returning citizens.

## Data Availability

The data generated during this study is not publicly available. All data were collected as part of a quality improvement initiative and not approved by the U.S. Department of Veteran Affairs to share.
